# Medical and Physical Intelligence

**Published:** 1823-08

**Authors:** 


### MEDICAL AND PHYSICAL INTELLIGENCE.

### ANATOMY.

1. *Malformation in a Male Infant.*—Mr. ASHFORD, of Hinckley, has favoured us with the following case:—Mrs. Holtham, of that place, was delivered, in June last, of twins: the first child, a female, was born perfect: the second, a male, lived about six weeks. It was observed that the umbilicus was but a short distance above the pubes, immediately under which, upon the abdominal surface, terminated the ureters. The vesica urinaria was wanting; the glans penis was exposed, and resembled a cone flattened on one side; there was no appearance of urethra. The testicles were situated on each side, just behind Poupart's ligament, and were in themselves seemingly perfect.

2. *Extraction of the Skeleton of a Fœtus.*—The case of Elizabeth Aterwell, mentioned in a late Number of this Journal, is now nearly brought to a successful termination. Mr. GUNNING has extracted the whole of the skeleton of the fœtus, with the exception of one clavicle. The shape and size of the bones of the cranium made it necessary to enlarge the wound upon more than one occasion, for the tendency to heal was with difficulty restrained. The bones are now in the possession of the College of Surgeons, and we understand that Mr. CLIFT has bestowed much pains in arranging them.

### PATHOLOGY.

3. *Spontaneous Combustion.*—MM. COLSON and LELAYE describe a case of spontaneous combustion, which they were ordered to examine by the legal authorities, on the 22d January, 1822. It occurred in the person of the Sieur Vatin, formerly a brewer, aged upwards of sixty years, and who had retired and lived for some time in a state of complete inactivity. The continued abuse of spirituous liquors had so affected his intellects as to dispose him, together with a cancerous ulcer occupying the left side of the face, to the commission of suicide, which he had frequently threatened. He had already some time before attempted to suffocate himself with the vapour of charcoal. He was of a sanguineo-lymphatic temperament, very bulky and tall, and, excepting the above-named ulcer, enjoyed good health. The evening prior to his death he had passed with his friends; and a woman, who lived in the house, was certain that at midnight he had gone to bed, and extinguished his light. At eight o'clock the next morning, a thick smoke, issuing from the room, raised suspicion in the neighbours; who, finding the door fastened within, broke it open, and saw the dead body lying on the floor, consumed by a flame, which they extinguished with difficulty, by pouring over it a great quantity of water. The weather was fine, and the thermometer somewhat below the zero. The room was on the ground-floor, spacious, and with a window looking towards the east.

MM. Colson and Lelaye found the body on the floor, a few feet distant from the bed; a chair, the straw and wood-work of which were burnt, was overturned in the direction of the body, near an iron cauldron, containing a small quantity of charcoal partly consumed: the water poured into this contained a good deal of fat. The head was attached to the neck, the flesh of which, both behind and at the sides, was destroyed; the face was swollen, and of a dark-red colour, as is observed in those who die from suffocation. On the left side, the parietes of the chest, and the whole upper extremity, were consumed, and only carbonised portions of the ribs and humerus were found; and, on the right side, the posterior portions of the ribs, the shoulder, and the arm. The hand, bent upon the epigastrium, was destroyed, as well as part of the fore-arm, and the transverse apophysis of the dorsal vertebræ

on the left side. Of the viscera, the lungs, heart, liver, and spleen, were torrefied, though preserving their form, and, when cut into, affording no blood. There were no vestiges of the other viscera. Other parts of the body were less burned. In the room nothing was burnt, except the charcoal, which had been alight in the cauldron, and which he had purchased the evening before.

MM. Colson and Lelaye ask, how it is possible to account for the destruction of a body of such a size, and in so short a time, otherwise than by supposing that, by intemperance, his organs had acquired the singular property of being destroyed by the contact of an ignited body. —(*Journal Complementaire,* June.)

### THERAPEUTICS.

4. *Helminthology.*—M. Virey informs us that he has received a memoir on the vermifuge powers of the pomegranate bark (*punica granatum,*) from Dr. Gomez, who relates fourteen cases of its successful exhibition. Dr. G. presents us also with descriptions of five varieties of tænia, different from those found in the human body in our climate. It is known, in fact, that entozoaries are not the same in all countries, but that they appear to have a preference: the *tænia lata,* or *botheriocephalus latus,* of Rudolphi, is most frequent in the northern regions; the *cucurbitana* is met with especially in Italy, according to Brera; and it is to be presumed that the *filaria papillosa* of Rudolphi is one of the causes of the ophthalmia of Egypt, as a pellucid ascaris has been found in the vitreous humour of the eyes of horses in India. If the figures and description given by M. Gomez are exact, containing the forms of the heads of five tænia he has observed, the catalogue of the species that attack mankind must be augmented. It will be especially necessary with respect to that tænia (No. 6,) which has five orifices in the head,—one central, and four surrounding it. The following are its distinguishing characters:

Pentastoma, capiti oculis quinque munito, articulis brevibus, poro laterali alternante, corpore gracili. Esp. Pentastoma coarctata, articulis alternatim poro laterali unico munitis.—(*Jour. de Pharm.* Mai.)

### MIDWIFERY.

5. *Rupture of the Umbilical Cord.*—M. Chaussier and others having doubted the possibility of the rupture of the umbilical cord by the mere weight of the child, M. A. P. Meirieu presents us with the case of a Madame P., thirty-five years of age, in good health, and pregnant with her third child, who was struck by the pole of a coach on the left side of the abdomen, in the eighth month of her pregnancy. She, however, was not taken in labour until the conclusion of the ninth month, and was brought to bed of a female child. A few moments before this event, Madame P. was walking about her room, and was seized with a strong pain; she took firm hold of the bed-post, brought herself nearer to the ground, retained the infant by means of her clothes, and placed it gently on the floor: the whole was the affair of an instant. One of the assistants took the child, and found the umbilical cord broken. This was before the arrival of Dr. Meirieu, who, on examination, found not the slightest traces of contusion on the child: it had, however, a spina bifida, occupying the lower part of the loins and the upper part of the sacrum. The umbilical cord was separated about four inches from the ring, and the end drawn out to a point.—(*Journal Universel*, Mai.)

MISCELLANEOUS.

6. *College of Surgeons.*—At the annual election of the officers of this College, which took place on the 22d of July, Henry Cline, Esq. was elected president, and Wm. Norris, Esq. and Sir Ludford Harvey, vice-presidents. It should be universally known that, in the Act lately passed for consolidating the laws relating to jails and houses of correction, to which the magistrates have the power of appointing surgeons, it is enacted that the surgeons must be *members of the Royal Colleges of Surgeons.* Correct lists of the London College may be obtained, free of expence, by magistrates, the clergy, and gentlemen, residing in the country, by application to the secretary of the College in Lincoln's-Inn Fields.

7. *Annual Report of the General Committee of the Associated Apothecaries and Surgeon-Apothecaries of England and Wales, received and adopted at the annual General Meeting of the Association, held at the Crown and Anchor Tavern, Strand; July 2d,* 1823.—Joseph Hayes, Esq. President.

Your Committee, in compliance with annual custom, submit to the Association the result of their proceedings since the last general meeting.

The meetings of the committee have been regularly held, although there has been but little business to transact, and that little of no particular interest or urgency.

The committee have not, however, been indifferent to the objects for which the Association was formed, and in the first volume of Transactions, now nearly completed, although necessarily partaking of the imperfections incident to the commencement of a new undertaking, they trust will be found an earnest of the zeal which they entertain for the welfare of the medical profession, and of their sincere desire to render the exertions of its members subservient to the public good and the best interests of humanity.

Viewing, as they do, with satisfaction the success which has attended, so far, the exertions of this Association in the improved state of medical education, particularly in that of the general practitioner, they still hope to witness further benefits, which cannot ultimately be otherwise than reciprocal between the profession and the public.

Well aware of the difficulties which attended the passing of the Apothecaries' Act, and of the opposing obstacles which prevented the fulfilment of the laudable wishes of the Association with which it originated, and of the profession at large, in rendering it fully adequate to its proposed end, the committee still cherish the hope of obtaining further improvements through the medium of legislature, which, whilst they may afford the necessary protection of the various members of the profession, shall be founded on a more dignified and extensive basis, the public good.

They therefore abstain, in the present instance, from any other than general allusion to the imperfections of the existing state of the medical profession, and of the legislative provisions relative to it, or the means required to effect the necessary ameliorations. The work of improvement is slow and laborious; but, when attempted with zeal, and carried on with perseverance and a purity of motive, which the end desired must show to be above suspicion, it cannot be otherwise than progressive.

They who now labour to place the practice of medicine upon a footing more consistent with its real dignity and usefulness, can scarcely expect personally to reap any benefit, unless the consciousness of having strenuously exerted themselves to effect the diminution of human suffering can be supposed to bear such an interpretation.

As an accurate knowledge of the present state of the medical profession can be the only ground-work of any rational attempt to effect improvement, the committee beg leave to recommend to the Association that it be therefore an instruction to the future committee—

1st. To ascertain, as far as may be practicable, the actual condition of the medical profession, and the obstacles which have hitherto impeded its advancement.

2d. To examine and arrange the imperfections to which its present condition is liable, showing the particulars in which the public and members of the profession are injured or aggrieved.

3d. To point out the remedies for the evils complained of.

4th. To use such means as to them may seem proper to effect (whether by application to the legislature or by other legal means,) the ameliorations to be desired.

5th. To continue to collect and record the experience of the profession on subjects relating to medical science, and to publish the same in occasional or annual volumes, calculated to prove the just claims of the different classes of the practitioners of the healing art to that confidence of the public which they have so long enjoyed.

6th. To give a brief account of the discoveries and improvements which have been made in medicine, surgery, and the accessory sciences, within the preceding year.

7th. To offer such encouragements of honorary rewards as may to them (the committee) seem proper, for the best practical Essays on select subjects relating to medicine and surgery, as may call forth the energies, particularly of the younger members of the profession, and elicit improvements in the healing art.

8th. That the profits arising from the publication of the Transactions of this Association be devoted to the above laudable purpose.

(Published by order of the meeting)

1, *Keppel-street, Bedford-square.* JOHN POWELL, *Secretary.*

8. *Army Medical Officers' Insurance Society and Benevolent Fund.*—The eighth anniversary of these institutions was celebrated on the 26th of May, at the Thatched-house Tavern, Sir E. HOME in the chair; when the directors-general and nearly one hundred medical officers sat down to dinner, besides many of their professional brethren: among others, the presidents of the Colleges of Physicians and Surgeons, and the master of the Apothecaries' Company, Drs. Baillie, &c. &c. It must be gratifying to such of our readers as are members of these most excellent societies, to learn the flourishing condition of their funds; that of the former amounting to £28,600, and affording an annual income from subscription *alone* of £1,700 10*s.* 9*d.*; that of the latter amounting to £2,000, having during the last year received an addition of £600. The utility of these establishments is too obvious to require comment; and we shall only add our wishes that their prosperity may continue to increase.

9. *Improved Truss.*—In a former Number, we mentioned the truss invented by Mr. COLES: the following certificate tends strongly to confirm our favourable opinion of the utility of this invention.

" I hereby certify, that Mr. Coles, of London-bridge, has perfectly succeeded in keeping up a very difficult and complicated rupture, on which I operated some years since; in which case many of the best truss-makers in London had failed to effect any relief. When I placed this patient under his care, I promised, should he succeed, to give him a certificate of my approbation, and, in conformity with that, I now state my firm conviction that his truss will be found more efficacious than any at present employed in similar cases. I have since tried his trusses in other cases, and have been much pleased with the ingenuity he has displayed in adopting his means to the circumstances of each case. HENRY EARLE."

" *George-street, Hanover-square; July* 23, 1823."

---

### METEOROLOGICAL JOURNAL,

*From June* 20, *to July* 19, 1823.

By Messrs. William Harris and Co. 50, Holborn, London.

| | MOON. | Rain gauge | THERM. | | | BAROM. | | DE LUC'S HYG. | | WIND. | | ATMOSPHERIC VARIATION. | | |
|---|---|---|---|---|---|---|---|---|---|---|---|---|---|---|
| | | | 9 A.M. | MAX. | MIN. | 9 A.M. | 10P.M. | 9 A.M. | 10P.M. | 9 A.M. | 10P.M. | 9 A.M. | 2 P.M. | 10P.M. |
| June | | | | | | | | | | | | | | |
| 20 | | | 60 | 70 | 55 | 29.84 | 29.80 | 67 | 70 | N | N | Fine | Fine | |
| 21 | | | 55 | 64 | 50 | 29.76 | 29.90 | 70 | 69 | N | N | Overc. | Overc. | Overc. |
| 22 | | | 52 | 55 | 49 | 29.85 | 29.87 | 67 | 68 | N | N | | | |
| 23 | ○ | | 59 | 62 | 54 | 29.74 | 29.72 | 68 | 67 | W | WNW | Cloud. | Fine | |
| 24 | | | 56 | 64 | 55 | 29.70 | 29.70 | 69 | 68 | SW | SW | Fine | | |
| 25 | | | 57 | 62 | 57 | 29.64 | 29.50 | 70 | 70 | N | SW | Fine | | Sho'ry |
| 26 | | .03 | 60 | 68 | 61 | 29.44 | 29.34 | 60 | 73 | SW | SE | Fine | Sho'ry | Sho'ry |
| 27 | | .05 | 57 | 67 | 57 | 29.20 | 29.11 | 73 | 73 | E | SSW | Overc. | Th. R. | Rain |
| 28 | | .09 | 59 | 67 | 60 | 29.14 | 29.25 | 75 | 78 | SSW | SW | Overc. | Rain | |
| 29 | | | 65 | 68 | 55 | 29.46 | 29.53 | 78 | 80 | SW | SW | Cloud. | Th. R. | Fine |
| 30 | | .02 | 62 | 67 | 60 | 29.74 | 29.68 | 74 | 76 | SW | S | Fine | Cloud. | Overc. |
| July | | | | | | | | | | | | | | |
| 1 | ☾ | | 65 | 68 | 61 | 29.71 | 29.80 | 79 | 80 | NNE | SSE | Cloud. | Rain | Cloud. |
| 2 | | | 66 | 70 | 56 | 29.80 | 29.83 | 77 | 78 | SSW | E | Fine | | |
| 3 | | | 67 | 68 | 57 | 29.84 | 29.90 | 70 | 78 | NNE | NNE | Cloud. | Fine | |
| 4 | | | 60 | 62 | 58 | 29.83 | 29.77 | 79 | 79 | SSE | SSW | Rain | Rain | Cloud. |
| 5 | | | 61 | 68 | 60 | 29.76 | 29.70 | 78 | 80 | SSW | SSW | Fine | | |
| 6 | | | 62 | 68 | 60 | 29.70 | 29.54 | 77 | 78 | SSW | SSW | Cloud. | | |
| 7 | | | 61 | 65 | 53 | 29.50 | 29.50 | 75 | 79 | SW | WSW | Cloud. | Rain | Cloud. |
| 8 | ● | .47 | 63 | 64 | 49 | 29.50 | 29.53 | 73 | 76 | WSW | W | Cloud. | Fine | |
| 9 | | | 60 | 65 | 49 | 29.73 | 29.80 | 67 | 68 | W | WSW | Fine | | Fine |
| 10 | | | 59 | 63 | 50 | 29.86 | 29.75 | 60 | 61 | WSW | SSE | | | Cloud. |
| 11 | | | 60 | 70 | 56 | 29.54 | 29.52 | 59 | 59 | S | SSE | | | Fine |
| 12 | | | 60 | 69 | 52 | 29.50 | 29.55 | 57 | 55 | SSW | SSW | Cloud. | | Cloud. |
| 13 | | | 59 | 64 | 54 | 29.58 | 29.54 | 54 | 67 | S | SSW | Cloud. | Rain | Rain |
| 14 | | | 61 | 65 | 54 | 29.50 | 29.60 | 53 | 54 | SW | SW | Fine | | Fine |
| 15 | ☽ | .29 | 60 | 63 | 55 | 29.60 | 29.40 | 51 | 53 | SSW | SSW | | | Rain |
| 16 | | .21 | 59 | 64 | 50 | 29.41 | 29.40 | 50 | 57 | SSW | SW | | Sho'ry | H. Ra. |
| 17 | | .40 | 60 | 63 | 55 | 29.64 | 29.72 | 54 | 56 | W | NW | | Fine | Fine |
| 18 | | | 61 | 62 | 57 | 29.60 | 29.63 | 80 | 85 | SSE | W | Rain | Cloud. | Rain |
| 19 | | | 60 | 65 | 58 | 29.75 | 29.72 | 75 | 78 | SSW | SW | Cloud. | Rain | Cloud. |

The quantity of rain during the month of June, was 1 inch and 25.100ths.

